# Robotic right hemicolectomy for transverse colon cancer in a patient with stringer type IIa intestinal malrotation: a case report with intraoperative video

**DOI:** 10.3389/fsurg.2026.1895886

**Published:** 2026-08-27

**Authors:** Go Ito, Dai Shida, Jun Imaizumi, Junko Mukohyama, Satoko Monma, Naoki Sakuyama

**Affiliations:** Division of Frontier Surgery, The Institute of Medical Science, The University of Tokyo, Tokyo, Japan

**Keywords:** anatomical variation, colon cancer, intestinal malrotation, pure duodenal non-rotaion, robotic right hemicolectomy

## Abstract

**Background:**

Intestinal malrotation results from disrupted midgut rotation and fixation during embryogenesis. Pure duodenal non-rotation (Stringer Type IIa), featuring isolated duodenal malrotation with a normally positioned colon, is extremely rare. Its unique anatomical distortions—specifically the absence of standard duodenal landmarks—present significant challenges for colorectal surgery.

**Case description:**

An asymptomatic 61-year-old male was diagnosed with transverse colon cancer during routine screening. Preoperative three-dimensional computed tomography revealed Type IIa malrotation: a vertically descending duodenum lacking the horizontal portion and a reversed distal relationship between the superior mesenteric artery and vein. We performed a robotic-assisted right hemicolectomy. To navigate the altered anatomy safely, we utilized a lateral-to-medial approach, providing an early anatomical roadmap before vascular dissection. The robotic platform's high-definition visualization facilitated precise identification of distorted vasculature and safe D3 lymphadenectomy. Pathological examination confirmed pT3N0M0, Stage IIA. The postoperative course was uneventful except for a minor surgical site infection; the patient remains recurrence-free at 3-year follow-up.

**Conclusion:**

Pure duodenal non-rotation presents unique challenges, including loss of the duodenal C-loop landmark and mesenteric vascular reversal. Preoperative recognition through detailed imaging is essential for safe surgical planning. This case demonstrates that robotic surgery is safe and efficient in complex settings via a strategic lateral-to-medial approach. The accompanying video provides a valuable resource for managing this rare anomaly.

## 1 Introduction

Intestinal malrotation encompasses a spectrum of congenital anomalies arising from the disruption of the normal 270-degree counterclockwise rotation and subsequent fixation of the midgut during embryogenesis (1, 2). While various classification systems exist, the framework described by Stringer remains the most widely utilized (3). Under this system, malrotation is categorized into three primary types: Type I (non-rotation), where both the duodenum and colon remain unrotated; Type II (incomplete rotation), characterized by an arrest of rotation at intermediate stages; and Type III (incomplete fixation), where rotation occurs normally but mesenteric attachment is deficient, predisposing patients to volvulus (4).

Within this classification, Type IIa—characterized by isolated duodenal non-rotation while the colon exhibits relatively normal positioning—represents an exceptionally rare subtype. Although malrotation is predominantly a pediatric condition (5, 6), a subset of patients remains asymptomatic until adulthood, where it is often discovered incidentally during imaging for unrelated pathologies (7). Due to its rarity, the true incidence of Type IIa malrotation remains unknown, and its unique anatomical implications for adult colorectal surgery have not been fully elucidated.

The altered anatomical relationships inherent to malrotation—specifically the absence of standard duodenal landmarks and the presence of mesenteric vascular anomalies—present significant challenges for surgical planning. Accurate identification of these distorted structures is pivotal for performing a safe and oncologically adequate dissection. In recent years, **robotic surgery** has gained increasing acceptance in colorectal oncology due to its advantages of high-definition three-dimensional (3D) visualization and precise maneuverability (8, 9). These technological features may be particularly beneficial in navigating the complex anatomical variations associated with Type IIa malrotation, where depth perception and fine instrumentation are essential for safe vascular management.

To date, reports of robotic colorectal surgery in patients with intestinal malrotation remain scarce. To our knowledge, the present case is the first to describe a robotic right hemicolectomy specifically for pure duodenal non-rotation (Type IIa). Herein, we report this unique case with an accompanying intraoperative video (Supplementary Video 1). This report aims to describe the specific anatomical challenges encountered, discuss the surgical strategies for this rare condition, and review the relevant literature to provide a practical resource for surgeons managing similar congenital anomalies.

## 2 Case presentation

### 2.1 Clinical features and preoperative evaluation

A 61-year-old male was referred to our department following the incidental finding of abnormal uptake in the transverse colon on positron emission tomography–computed tomography (PET-CT) during a comprehensive health screening. The patient was completely asymptomatic, denying abdominal pain, altered bowel habits, or rectal bleeding. His medical history was notable for an appendectomy at age 15, a facial nerve palsy, and a right internal carotid artery aneurysm. Physical examination and preoperative laboratory investigations, including complete blood count and biochemistry, were unremarkable. Serum tumor markers were within normal limits (CEA: 1.7 ng/mL, CA19-9: 6.9 U/mL).

**Figure 1:**
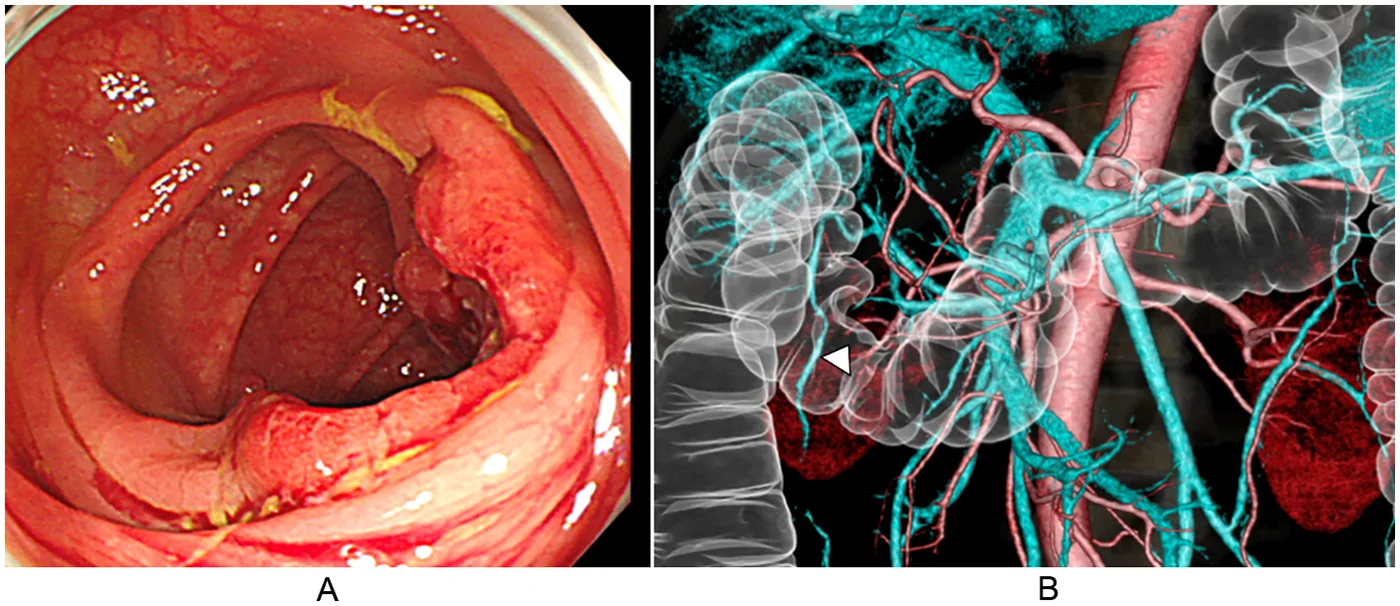
Endoscopic and radiological findings of the tumor. **(A)** Colonoscopy revealed a semicircumferential, circumscribed ulcerative lesion measuring 4 cm in diameter in the proximal transverse colon, causing significant luminal narrowing. **(B)** Three-dimensional (3D) reconstructed CT image demonstrating the tumor in the proximal transverse colon (white arrowhead).

Colonoscopy revealed a semicircumferential, circumscribed ulcerative lesion measuring 4 cm in diameter in the proximal transverse colon (Figure 1A). Biopsies confirmed a well-to-moderately differentiated adenocarcinoma. Three-dimensional (3D) reconstructed CT imaging correlated with the endoscopic findings, demonstrating the tumor in the proximal transverse colon (Figure 1B).

### 2.2 Anatomical assessment of intestinal malrotation

**Figure 2:**
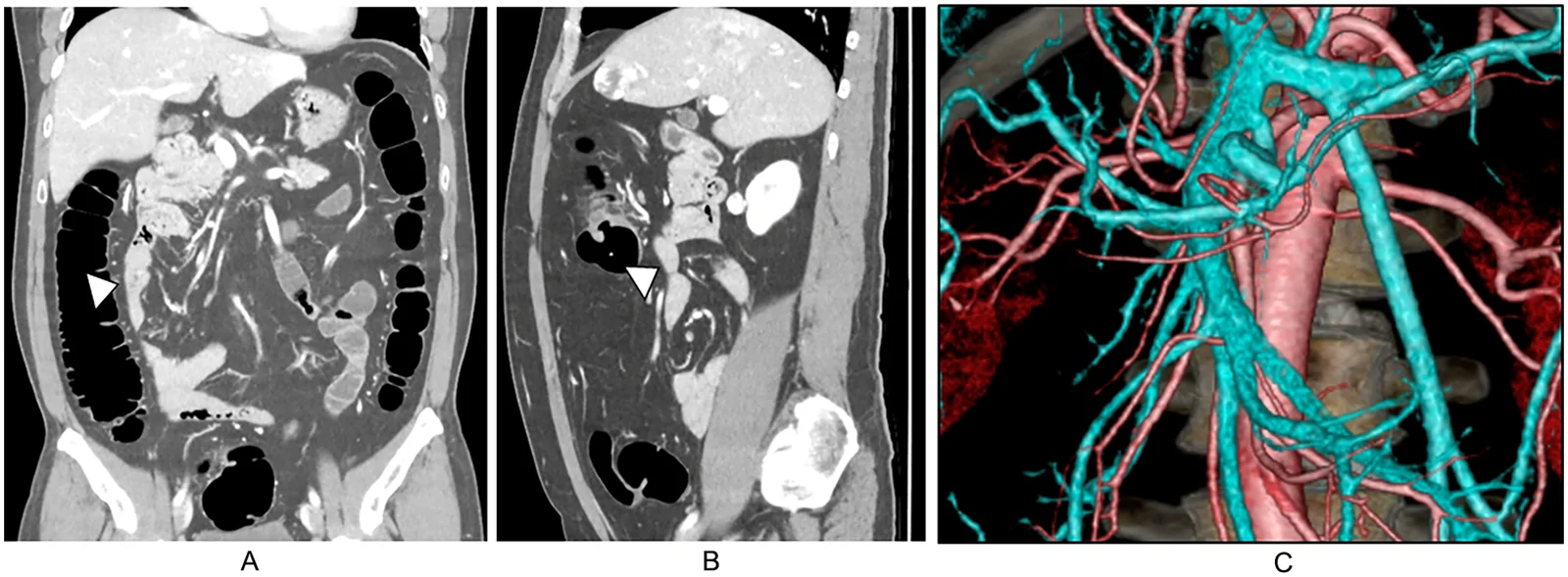
Preoperative radiological evaluation of the anatomical anomalies. **(A,B)** Axial contrast-enhanced CT images showing duodenal non-rotation. The duodenum (white arrowhead) descends linearly along the right side of the pancreatic head, failing to form a C-loop or cross anterior to the abdominal aorta. **(C)** Three-dimensional volume-rendered reconstruction of the mesenteric vasculature, illustrating the reversed anatomical relationship between the superior mesenteric artery (SMA) and the superior mesenteric vein (SMV).

Contrast-enhanced CT showed semicircumferential wall thickening of the proximal transverse colon without evidence of extramural extension or distant metastasis. Crucially, a meticulous radiological review identified significant anatomical anomalies consistent with Stringer Type IIa malrotation (Figure 2):

1. Duodenal anomaly: The duodenum descended vertically along the right side of the pancreatic head. The third (horizontal) portion was entirely absent; thus, the duodenum failed to cross the midline ventral to the abdominal aorta (Figures 2A, B).
2. Mesenteric vascular relationship: While the superior mesenteric vein (SMV) was positioned to the right of the superior mesenteric artery (SMA) at their origin, a reversed relationship was observed distally, where the SMA crossed over to the right of the SMV (Figure 2C).

Based on these findings—isolated duodenal non-rotation with a normally positioned colon—a preoperative diagnosis of pure duodenal non-rotation (Type IIa) (3) was established.

### 2.3 Robotic surgical procedure

Given the complex vascular and duodenal anatomy, we performed a robotic-assisted right hemicolectomy using the da Vinci Xi surgical system (10). We hypothesized that the robotic platform's high-definition 3D visualization and wristed instrumentation would provide a significant advantage in navigating the altered anatomy.

**Figure 3:**
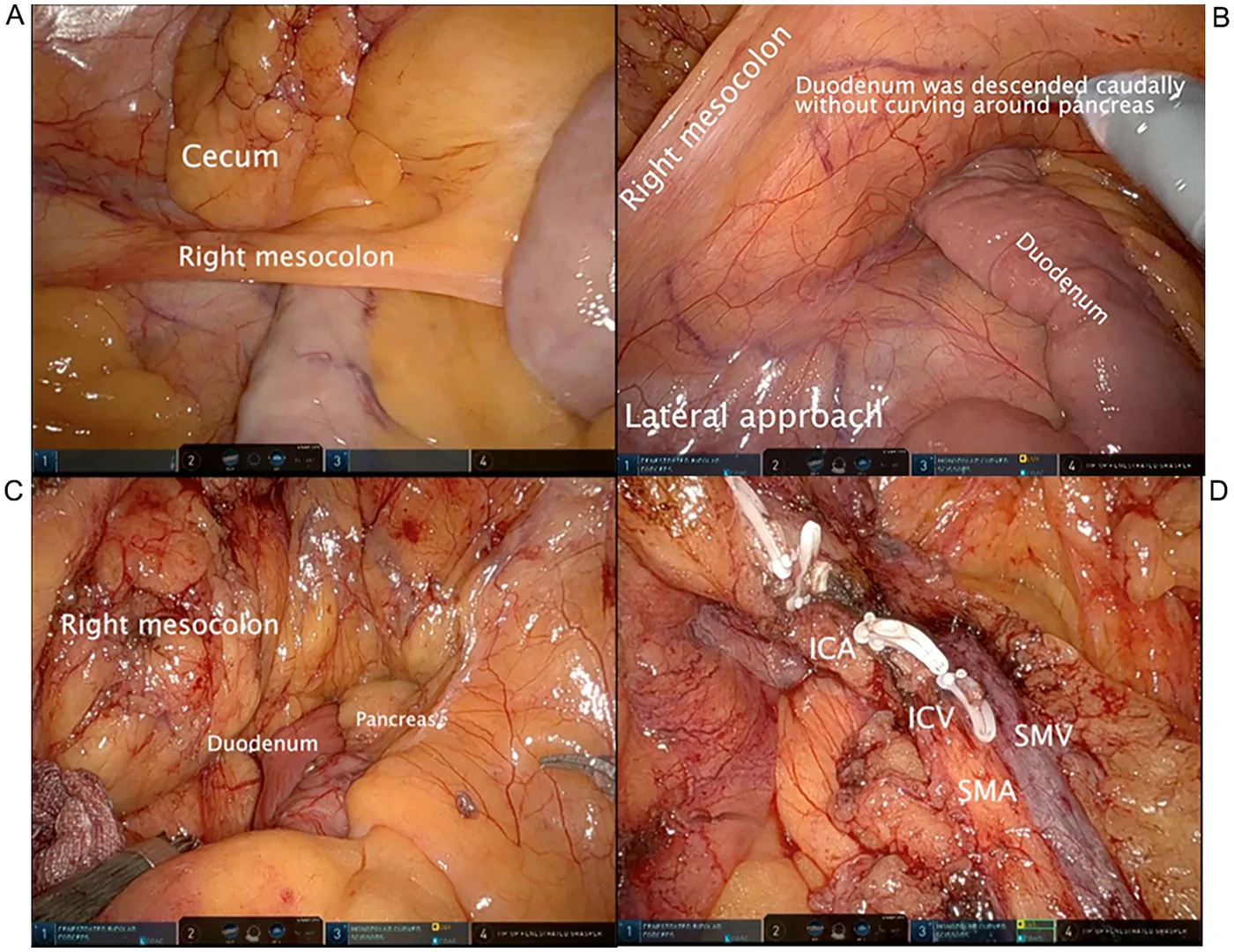
Intraoperative findings during robotic right hemicolectomy**. (A)** Initial view showing the ascending colon fixed to the lateral abdominal wall, with the small intestine situated dorsal to the right mesocolon. **(B)** Direct visualization of the duodenum descending caudally without curving around the pancreatic head. **(C)** View after completion of the lateral-to-medial mobilization, confirming the linear course of the duodenum and the complete absence of its third (horizontal) portion. **(D)** Final view of the medial approach, confirming the reversed SMA-SMV relationship at the distal mesenteric root.

Initial abdominal exploration confirmed the preoperative diagnosis. The ascending colon was fixed only to the lateral abdominal wall with a lack of normal embryologic fusion, and no Ladd's bands were identified (Figure 3A).

After docking the robotic system, the procedure commenced with a lateral-to-medial approach with prior dorsal mobilization. First, lateral mobilization of the right colon was performed by taking down the lateral peritoneal attachments. In this study, we adopted this specific approach, which is performed entirely within the peritoneal cavity. As a key surgical sequence, the right colon and the mesocolon are initially mobilized from the dorsal connective tissue; this prior mobilization facilitates the subsequent medial approach for D3 lymphadenectomy along the surgical trunk of the superior mesenteric artery. In the present case, lateral mobilization of the right colon was first performed by taking down the lateral peritoneal attachments. As anticipated from preoperative imaging, the duodenum did not curve around the pancreas to form the typical C-loop configuration (Figure 3B). Instead, it descended in a straight course toward the caudal direction along the right side of the pancreatic head, transitioning into the proximal jejunum without the characteristic horizontal segment that would normally cross anterior to the great vessels (Figure 3C).

Subsequently, a medial-to-lateral approach was initiated to perform the lymphadenectomy. In conventional right hemicolectomy, the third portion of the duodenum serves as the primary landmark for identifying the ileocolic pedicle. In this case, the absence of the horizontal segment necessitated an alternative strategy: the SMA and SMV were carefully traced from their origins to identify the ileocolic artery (ICA) and vein (ICV). The distal reversed relationship of the SMA/SMV was clearly visualized under 3D magnification, allowing for the safe ligation of the ICA and ICV at their respective roots (Figure 3D). The right branch of the middle colic vessels was also identified and divided (Supplementary Video 1).

The specimen was extracted through a small umbilical incision, and a functional end-to-end ileocolic anastomosis was performed extracorporeally. The total operative time was 417 min, with an estimated blood loss of 226 mL and no intraoperative complications.

### 2.4 Pathological findings and postoperative course

Macroscopic examination revealed a 40 × 25 mm ulcerative tumor in the proximal transverse colon. Histopathology confirmed a well-differentiated tubular adenocarcinoma, staged as pT3 N0 M0 (Stage IIA). Notably, 50 lymph nodes were retrieved, all of which were negative for malignancy.

The postoperative course was complicated only by a minor surgical site infection, which was managed conservatively. The patient was discharged on postoperative day 12. At the 3-year follow-up, the patient remains alive and disease-free, with no evidence of recurrence on surveillance imaging.

## 3 Discussion

We successfully performed a robotic right hemicolectomy for transverse colon cancer in a patient with Stringer Type IIa malrotation (pure duodenal non-rotation). This rare congenital variant, characterized by a vertically descending duodenum without a horizontal (third) portion and a normally positioned colon, presents unique intraoperative challenges—most notably the loss of the standard anatomical landmarks that surgeons rely on for orientation. Furthermore, the reversed distal relationship of the superior mesenteric artery (SMA) and superior mesenteric vein (SMV) observed in our case posed a significant risk of vascular misidentification.

In this complex anatomical setting, the robotic platform facilitated the safe and efficient execution of the planned oncological resection. The high-definition three-dimensional (3D) visualization and magnified operative field allowed for the precise, “skeletonized” identification of the mesenteric vasculature, effectively compensating for the lack of a normal duodenal C-loop. The stable, multi-degree-of-freedom instrumentation enabled meticulous D3 lymphadenectomy despite the proximity of the SMA to the operative field. These technological features, combined with rigorous preoperative planning, allowed us to overcome the inherent anatomical hurdles and perform the procedure safely.

Right-sided colon cancer associated with Type IIa malrotation is exceptionally rare, with only two cases previously reported (4, 11). Notably, in one of these reports—a laparoscopic case—the authors remained unaware of the Type IIa classification even at the time of publication (11). However, their intraoperative findings are highly pathognomonic for this specific variant. Specifically, the authors reported that an initial medial approach resulted in anatomical disorientation, a failure to identify Toldt's fusion fascia, and inadvertent penetration of the mesocolon, along with hypoplasia of the ligament of Treitz. This underscores the fact that Type IIa variants can be easily overlooked intraoperatively if the surgeon is not specifically looking for them. Such a lack of recognition inevitably leads to a loss of intraoperative orientation, causing significant surgical disorientation and potentially hazardous dissection. Therefore, it is of paramount importance for surgeons to be aware that Type IIa variants exist within the spectrum of malrotation—albeit rarely—and to proactively identify these anatomical hallmarks during preoperative imaging. We believe that a heightened awareness of this subtype, supported by the detailed anatomical insights provided by our accompanying intraoperative video (Supplementary Video 1), will serve as a valuable practical resource to aid surgeons in correctly identifying this anomaly and avoiding intraoperative disorientation.

To clearly delineate the surgical nuances of this rare anomaly compared to a standard robotic right hemicolectomy, several key anatomical landmarks and tactical differences must be highlighted (Table 1). In a standard procedure, the third (horizontal) portion of the duodenum serves as a reliable geometric landmark for identifying the surgical plane and locating the ileocolic pedicle. In the present case, the complete loss of this C-loop configuration, combined with the distal reversal of the SMA and SMV, posed a significant risk of vascular misidentification and surgical disorientation. Therefore, instead of relying on central landmarks via an initial medial approach, a comprehensive understanding of these differences is essential for planning a safe procedure.

**Table 1:** Comparison of anatomical characteristics and surgical strategies between standard right hemicolectomy and the present case with type IIa malrotation.

Anatomical/surgical features	Standard right hemicolectomy	Present case (stringer Type IIa malrotation)
Duodenal configuration	Standard C-loop with a horizontal (3rd) portion crossing the midline anterior to the aorta.	Vertically descending duodenum without a horizontal portion; fails to cross the midline.
Primary surgical landmark	The 3rd portion of the duodenum guides the mesocolic-retroperitoneal plane and ileocolic pedicle.	Absent. The superior mesenteric vessels must be traced from their origins to identify the pedicle.
SMA/SMV relationship	The SMV is consistently located to the right side of the SMA.	Reversed distal relationship; the SMA crosses over to the right side of the SMV.
Optimal surgical sequence	Medial-to-lateral approach is generally preferred for early vascular ligation (D3 lymphadenectomy).	Lateral-to-medial approach with prior retroperitoneal mobilization to establish a safe roadmap first.

Furthermore, our experience demonstrates that the sequence of the surgical approach is paramount in managing this rare anatomical variant. In the present case, we initiated the procedure with a lateral-to-medial approach with prior dorsal mobilization. This strategy allowed for early confirmation of the abnormal fixation of the right colon and the linear course of the duodenum before committing to major vascular dissection (Supplementary Video 1). By contrast, Yoshida et al. (11) initially attempted a medial approach without recognizing the duodenal anomaly, which led to the misidentification of the mesocolon and subsequent anatomical disorientation. Their eventual recommendation—to commence with a lateral approach—is strongly supported by our findings. Establishing the posterior plane of the right mesocolon and confirming the duodenal position laterally provides a clearer “roadmap.” This allows the surgeon to perform the subsequent medial dissection and lymphadenectomy with greater confidence and safety, particularly in the distal mesenteric region where vascular anomalies are most confounding. From a pediatric surgical perspective, the primary objective when managing intestinal malrotation is to prevent midgut volvulus and bowel obstruction by lysing Ladd's bands, broadening the narrow mesenteric base, and placing the bowel in a non-rotated configuration (Ladd's procedure). In contrast, when encountering an asymptomatic malrotation in an adult patient with right-sided colon cancer, the primary surgical goal shifts toward achieving complete mesocolic excision with D3 lymphadenectomy and obtaining negative surgical margins. In the present case, no obstructive Ladd's bands were identified. By adopting a systematic lateral-to-medial mobilization, we were able to safely flatten and broaden the mesocolon while directly visualizing the SMA trunk, thereby satisfying both oncologic requirements and the embryologic principles of mesenteric mobilization without the need for additional corrective procedures.

This case contributes to the limited literature on minimally invasive surgery for colorectal cancer in the context of congenital midgut anomalies. It demonstrates that with meticulous preoperative assessment, particularly the use of 3D-CT reconstructions and a strategically planned approach, robotic right hemicolectomy is a safe and robust tool for navigating the anatomical complexities of Type IIa intestinal malrotation. While the findings from a single case cannot be generalized and further accumulation of cases is necessary to establish the long-term oncological outcomes and the optimal surgical approach for this rare condition, our experience highlights a reproducible strategy for managing such challenging anatomical variations.

## 4 Conclusion

We report a rare case of transverse colon cancer associated with pure duodenal non-rotation (Type IIa) that was successfully managed with robotic right hemicolectomy. This rare congenital anomaly presents unique anatomical challenges, particularly the absence of standard duodenal landmarks and the presence of reversed mesenteric vascular relationships. Our experience underscores the critical importance of meticulous preoperative imaging, especially three-dimensional CT reconstruction, to accurately identify these variations and facilitate careful surgical planning.

While the altered anatomy requires a cautious intraoperative approach, this case demonstrates that robotic surgery can be performed safely and efficiently in such complex settings. Furthermore, the accompanying intraoperative video provides a detailed visual reference of the altered anatomy and our surgical maneuvers, which we believe will serve as a valuable practical resource for surgeons encountering similar anatomical challenges. Further accumulation of cases is warranted to establish standardized surgical strategies for this rare anatomical variant.

## Data Availability

The original contributions presented in the study are included in the article/Supplementary Material, further inquiries can be directed to the corresponding author.

## Supplementary material

Supplementary Video 1 **Intraoperative video of robotic right hemicolectomy for Type IIa malrotation**. The video highlights the unique anatomical challenges, including the identification of the non-rotated duodenum and the reversed mesenteric vasculature, managed via a strategic lateral-to-medial approach.
